# Staquorsin: A Novel *Staphylococcus aureus* Agr-Mediated Quorum Sensing Inhibitor Impairing Virulence *in vivo* Without Notable Resistance Development

**DOI:** 10.3389/fmicb.2021.700494

**Published:** 2021-07-05

**Authors:** Norhan H. Mahdally, Riham F. George, Mona T. Kashef, Medhat Al-Ghobashy, Fathia E. Murad, Ahmed S. Attia

**Affiliations:** ^1^Department of Microbiology and Immunology, Faculty of Pharmacy, Cairo University, Cairo, Egypt; ^2^Department of Pharmaceutical Chemistry, Faculty of Pharmacy, Cairo University, Cairo, Egypt; ^3^Department of Analytical Chemistry, Faculty of Pharmacy, Cairo University, Cairo, Egypt; ^4^School of Pharmacy, Newgiza University, Giza, Egypt

**Keywords:** *S. aureus*, quorum-sensing, resistance, Agr, MRSA, savirin, anti-virulence, anti-biofilm

## Abstract

The emergence of microbial resistance to the available antibiotics is a major public health concern, especially with the limited rate of developing new antibiotics. The utilization of anti-virulence agents is a non-conventional approach that can be used to combat microbial infection. In *Staphylococcus aureus*, many virulence factors are regulated by the Agr-mediated quorum sensing (QS). We developed a chemical compound that acts a potential Agr-inhibitor without reducing bacterial viability. The compound was designated staquorsin for S*taphylococcus aureus* QS inhibitor. *In silico* analyses confirmed the binding of staquorsin to the AgrA active site with an absolute binding score comparable to savirin, a previously described AgrA inhibitor. However, staquorsin turned out to be superior over savarin in not affecting the *S. aureus* viability in concentrations up to 600 μM. On the other hand, savirin inhibited *S. aureus* growth in concentrations as low as 25 μM. Moreover, staquorsin proved to be a potent inhibitor of the Agr system by inhibiting hemolysins, lipase production, and affecting biofilms formation and detachment. On the molecular level it significantly inhibited the effector transcript RNA III. *In vivo* testing, using the murine skin abscess model, confirmed the ability of staquorsin to modulate *S. aureus* virulence by effectively controlling the infection. Twenty passages of *S. aureus* in the presence of 40 μM staquorsin have not resulted in loss of activity as evidenced by maintaining its ability to reduce hemolysin production and RNA III transcript levels. In conclusion, we hereby describe a novel anti-virulence compound inhibiting the *S. aureus* Agr-system and its associated virulence factors. It is active both *in vitro* and *in vivo*, and its frequent use does not lead to the development of resistance. These findings model staquorsin as a promising drug candidate to join the fierce battle against the formidable pathogen *S. aureus*.

## Introduction

The continuous and widespread use of antimicrobial agents has generated a supreme environment for the selection of multidrug-resistant organisms (Chambers and Deleo, 2009). About 2.8 million people get infected by antibiotic resistant organisms, and more than 35,000 people die annually, just in the United States (CDC, 2019). In the developing countries, the situation could be more aggravated because of the flagrant abuse of antimicrobials (Ayukekbong et al., 2017). The rapid rate, at which resistant strains arise, is quite problematic in the medical community; given the slow rate of development of new antibiotics (Chambers, 2001). Therefore, new treatment approaches, with minimum chance of resistance development, must be sought (Totsika, 2016).

Anti-virulence agents represent a promising alternative to the use of antimicrobials. These compounds act by suppressing the organism’s virulence thus “disarming” it. As well as, reducing its ability to colonize and invade host tissue which enables host defense mechanisms to eradicate the infection (Munguia and Nizet, 2017). They should be designed to lack any inhibitory effect on the viability of the pathogen. This avoids the selection of resistant mutants; the problem which is threatening the continuation of antibiotic use in infection management. In addition, anti-virulence agents are tailor made against a specific target where their narrow spectrum will limit the opportunity for resistance development (Totsika, 2016).

Production of virulence factors is under the control of many regulatory mechanisms including the quorum sensing (QS). Microbial cells communicate where a certain cell density is required to activate the QS activity and other associated virulence factors. Inhibition of QS represents an extraordinary therapeutic target that disarms the pathogen from numerous virulence factors simultaneously (Miller and Bassler, 2001).

In *Staphylococcus aureus*, multiple virulence factors are under the regulation of the Agr-mediated QS. The *agr* operon contains two promoters: P2 and P3 that govern the production of two transcripts: RNA II and RNA III, respectively. The RNA II encodes proteins that are entangled in the QS activity (AgrB, AgrD, AgrC, and AgrA). The AgrB is responsible for the activation and production of the autoinducing peptide AgrD while the AgrC and AgrA represent a two-component regulatory system. When the extracellular autoinducing peptide concentration reaches a threshold, the Agr system is activated. Upon activation, the level of both transcripts and their proteins notably increases (Tan et al., 2018). The RNA III transcript encodes δ-hemolysin toxin and at the same time acts as a regulatory small RNA molecule (Le and Otto, 2015).

The Agr-system acts as a global regulator of virulence factors, both directly and indirectly. It activates the production of several extracellular toxins; α-, β-, and γ-hemolysins as well as lipases, leukotoxins, toxic shock syndrome toxins, and phenol soluble modulins. It can affect the expression of several virulence factors indirectly through transcription regulators such as Rot, SarT, and SarS. The *agr*-dependent virulence factors are important for the establishment of infection by promoting host colonization and invasion, and helping the escape from host defense mechanisms (Le and Otto, 2015).

**FIGURE 1:**
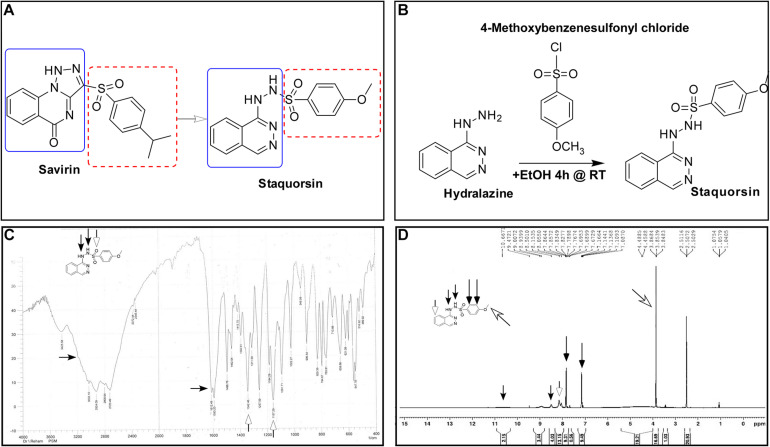
Synthesis and validation of staquorsin. **(A)** Rationale for the design of the staquorsin molecule as being an analogue for savirin. **(B)** The synthesis scheme used for obtaining staquorsin. **(C)** An IR spectrum chart for staquorsin. **(D)** A ^1^H NMR spectrum chart for staquorsin. In both charts, the peaks corresponding to the respective moieties are marked with same arrows labeling the chemical structure.

In this study, we have developed a new potent *agr*-inhibitor (staquorsin), which was aimed to be a new analog of savarin (*S. aureus* virulence inhibitor), that is a triazoloquinazoline derivative that inhibits AgrA (Gordon et al., 2013; Sully et al., 2014). The phthalazine nucleus was selected as a suitable substitute for the quinazoline ring of savirin (Figure 1A) as it is considered as its positional isomer. In addition, a cyclic-versus-non-cyclic replacement of the fused triazolo ring of savirin with the open hydrazine moiety (Figure 1A) is adopted. This is believed to produce a more flexible structure that may confer easier accommodation and interaction with the biological target. The action of this molecule is demonstrated on multiple levels including *in silico*, *in vitro*, and *in vivo* testing.

## Materials and Methods

### Ethics Statement

All procedures involving animals were approved by the Research Ethics Committee of the Faculty of Pharmacy, Cairo University with number MI (1682).

### Bacterial Strains and Culture Conditions

*Staphylococcus aureus* strain Newman (Duthie and Lorenz, 1952) was used as the wild-type strain in all the experiments. Its *agr*-mutant (*ΔagrB:erm*) (Bae et al., 2004) was used as a negative-control strain. USA300 strain was used as an example for a methicillin-resistant *S. aureus* (MRSA) strain (Diep et al., 2006). Cells were typically grown on tryptic soy agar (TSA) or in tryptic soy broth (TSB) with shaking at 180 rpm and incubated at 37°C.

### Chemical Compounds

4-Methoxy-N’-(phthalazin-1-yl) benzenesulfonohydrazide hydrochloride (staquorsin) was one among a group of other chemical compounds (George, 2018) screened for their *S. aureus* QS inhibitory activity. Staquorsin was selected for further analyses as it was the one showed the most promising results in a preliminary screening for anti-Agr activity. It was obtained according to the scheme described in Figure 1B. Briefly, equimolar amounts of hydralazine and 4-methoxybenzenesulphonyl chlorides (5 mmol) in 5 ml absolute ethanol were stirred at room temperature for 4 h. The obtained precipitate was filtered and washed with ethanol to give a creamy white solid. The structure was confirmed by both Infrared (IR) and proton nuclear magnetic resonance (^1^H NMR) spectroscopy techniques. The 3-[(4-isopropylphenyl) sulfonyl] [1,2,3] triazolo [1,5-a] quinazolin-5(4H)-one (savirin) was purchased from AK Scientific, Inc., United States. All chemicals were dissolved in dimethyl sulfoxide (DMSO) to a final concentration of 10 mM, aliquoted, and stored at −80°C.

### Molecular Docking to AgrA Active Site and the *in silico* ADMET Properties Prediction

All the molecular modeling studies were carried out using Molecular Operating Environment (MOE, 10.2008) software. All minimizations were performed with MOE until root mean square deviation (RMSD) gradient of 0.05 kcal⋅mol^–1^Å^–1^ with MMFF94x force field and the partial charges were automatically calculated. The X-ray crystallographic structure of the AgrA of *S. aureus* (PDB ID: 3BS1) was downloaded from the protein data bank http://www.rcsb.org/. Water molecules and ligands not involved in the binding were removed. Then the protein was prepared for docking study using *Protonate 3D* protocol in MOE with default options. Key amino acids Glu217, Arg218, Cys228, and Tyr229 were used to define the binding site for docking (Sully et al., 2014). Triangle Matcher placement method and London dG scoring function were used for docking. First, the well-known Agr-inhibitor “Savirin” was docked in the vicinity of the active site of the protein with the ability to reproduce all the reported interactions with the key amino acids in the binding site; hydrogen bonding with Arg218 and π–π stacking with the Tyr229 (Sully et al., 2014). The validated setup was then used in predicting the ligands receptor interactions at the active site for the tested compound staquorsin. The absorption, distribution, metabolism, excretion and toxicity (ADMET) of both savirin and staquorsin were conducted using the Swiss ADME www.swissadme.ch/(Daina et al., 2017).

### Assessment of the Alpha-Hemolysin Inhibition Activity

Cells were cultured in the presence of 40 μM of staquorsin for 22 h at 37°C in 5 ml TSB in a 50 ml falcon tube with shaking at 180 rpm. The hemolytic activity of the culture supernatant was determined as previously described (Bernheimer, 1988). Overnight cultures were normalized to the same OD_600_ then were centrifuged at 15,000 × *g*. The supernatants were serially diluted with sterile phosphate buffered saline (PBS) to a final volume of 600 μl and mixed with equal volume of freshly prepared rabbit RBCs suspension. The mixtures were incubated for 30 min at 37°C then centrifuged at 13,000 × *g* for 5 min. The absorbance of supernatant was measured at 540 nm and the HA_50_ was calculated as the reciprocal of the dilution that caused 50% hemolysis.

### Assessment of the Delta-Hemolytic Activity

The supernatants of the overnight cultures grown in the presence of 200 μM of staquorsin in conditions as described above were used to assess the delta-hemolytic activity as previously described (Russell et al., 2006). Briefly, wells with 10 mm diameter in sheep blood agar were filled with 50 μl of the filtered culture supernatant, then incubated for 24 h at 37°C, and the area of the clear zones formed in the plates were measured using AutoCAD (Autodesk, United States). The data was plotted as a percentage of the area of the positive control zone. Another measurement was performed after the plates were further incubated at 4°C for 24 h to exclude any interference from beta-hemolysin.

### Evaluation of the Lipolytic Activity

The supernatants of the overnight cultures used above for the delta-hemolytic activity were used to assess the lipolytic activity. This assessment was carried out according to the method described before (Smeltzer et al., 1992). Briefly, wells of 4 mm diameter in tributyrin agarose plates were filled with 20 μl filtered supernatants and the plates were incubated for 24 h at 37°C. The area of each zone was measured using AutoCAD and plotted as percentage of the area of the positive control.

### Assessing the Effect on Biofilms Formation and Detachment

In order to assess the role of staquorsin in biofilm formation and detachment pre- and post-exposure assays were performed as described before (Nour El-Din et al., 2020) with slight modifications. Briefly, for biofilm formation, overnight cultures of staphylococcal cells were normalized to OD_600_ of 1.0 and diluted 1:100 in fresh TSB. Then one hundred μL aliquots were mixed with equal volume to give a final conc. of 40 μM staquorsin, or equivalent amount of DMSO, in TSB and placed in the wells of a sterile, untreated polystyrene, 96-well flat-bottomed plate. The plates were incubated at 37°C without shaking for 24 h. OD_600_ of the overnight culture was measured and then wells were washed three times with PBS and dried overnight. Adherent cells were stained with crystal violet (0.4% w/v) at room temperature for 15 min. Then the wells were washed three times with PBS. Biofilm formation was quantified by adding 150 μL of absolute ethanol and incubating at room temperature for 30 min with mild shaking. Finally, a total of 125 μL of the eluate was then transferred to a new sterile polystyrene microtiter plate and the absorbance of the plate at OD_595_ was recorded. The biofilm formation ability was represented by dividing the crystal violet OD_595_ reading by the planktonic growth OD_600_ reading. For the biofilm detachment assay, biofilms were allowed to form as described above, then after 24 h, the OD_600_ of the wells was measured after the incubation for normalization purposes. Before the addition of the staquorsin, growth medium and the unattached cells were discarded, and the biofilms were washed twice with PBS. Next, fresh TSB with either 40 μM staquorsin, or equivalent amount of DMSO were added to the wells and incubated at 37°C for 24 h. Then the plates were processed as described above for biofilm formation.

### Quantitation of the RNA III Transcript

The level of the RNA III transcript was determined by quantitative reverse transcription polymerase chain reaction (qRT-PCR). The *S. aureus* cultures were incubated in the presence of 40 μM of staquorsin or equivalent volume of DMSO for 15 h at 37°C with shaking at 180 rpm (Eleaume and Jabbouri, 2004). RNA was isolated using the RNeasy mini kit (Qiagen, Germany) and reverse transcription was carried out by the QuantiTect Reverse Transcription Kit (Qiagen). The produced cDNA was quantified by real-time PCR using the GoTaq^®^ qPCR Master Mix (Promega, United States). The 16S rRNA transcript was used as a normalizer using the primer pair (5′-TGAGATGTTGGGTTAAGTCCCGCA-3′) and (5′-CGGTTTCGCTGCCCTTTGTATTGT-3′) (Attia et al., 2010). While for the RNA III transcript, the primer pair (5′-ATAGCACTGAGTCCAAGGAAA-3′) and (5′-GCC ATCCCAACTTAATAACCATGT-3′) was used. The data obtained were analyzed using the Rotor-Gene Q Software (Qiagen) applying the ^ΔΔ^ CT method (Livak and Schmittgen, 2001), and using the normalized transcript level of RNA III in the presence of DMSO as the calibrator.

### Determination of the MIC and Growth Inhibition Effect

The MIC, of both staquorsin and savirin, was determined by the broth microdilution method (CLSI, 2012), using a concentration range of 25–1600 μM. The MIC value was considered as the lowest concentration of the compound that showed no visible growth. In addition, to assess their possible effect on growth, the optical density of the cultures, was measured at 600 nm after 24 h of incubation at 37°C. Finally, in order to assess the effect of staquorsin on the growth kinetics of *S. aureus*, growth curves were constructed for both wild-type Newman and the MRSA strain USA300 in the presence of 40, 200, and 600 μM staquorsin. The growth pattern and extent were compared in the form of the growth curves to those of the same strains grown in the presence of the equivalent amounts of DMSO. Aliquots were taken at time points 0, 1, 2, 3, 4, 5, 6, 7, 8, and 24 h and the OD_600_ was recorded.

### *In vitro* Assessment of Possible Resistance Development

*Staphylococcus aureus* cells were incubated in the presence of 40 μM of staquorsin. After 24 h, the culture was centrifuged, and the pelleted cells were resuspended in the same volume of fresh TSB containing 40 μM of staquorsin and the whole process was repeated for 20 passages. Stocks were made from cells resulting from each passage. The alpha-hemolysin activity was assessed as described above for the cells obtained after 5, 10, 13, 15, 18, and 20 passages. Also, the relative concentration of RNA III was determined for the cells obtained after 15 and 20 passages. At the same time, erythromycin MIC against the wild-type *S. aureus* was determined. The wild-type strain was then passed in the presence of 0.8 μg/ml (sub-MIC) erythromycin, and erythromycin MIC was determined after 8 and 16 passages.

### Cytotoxicity Assay

Human skin fibroblast (HSF) was obtained from Nawah Scientific Inc., (Cairo, Egypt). Cells were maintained in Dulbecco’s Modified Eagle’s medium (DMEM) supplemented with 100 mg/mL of streptomycin, 100 units/mL of penicillin and 10% of heat-inactivated fetal bovine serum in humidified, 5% (v/v) CO_2_ atmosphere at 37°C. Cell viability was assessed by the sulforhodamine B (SRB) assay (Vichai and Kirtikara, 2006). Briefly, aliquots of 100 μL cell suspension (5 × 10^3^ cells) were placed in 96-well plate and incubated in complete media for 24 h. Cells were treated with another aliquot of 100 μL media containing staquorsin at various concentrations (0.01–100 μM). After 24 h of drug exposure, cells were fixed by replacing the media with 150 μL of 10% Trichloroacetic acid (TCA) and incubated at 4°C for 1 h. The TCA solution was removed, and the cells were washed five times with distilled water. Aliquots of 70 μL 0.4% w/v SRB solution were added and incubated in a dark place at room temperature for 10 min. Plates were washed three times with 1% acetic acid and allowed to air-dry overnight. Then, 150 μL of 10 mM TRIS were added to dissolve the protein-bound SRB stain. The absorbance was measured at 540 nm using a BMG LABTECH^®^- FLUOstar Omega microplate reader (Ortenberg, Germany).

### *In vivo* Evaluation of Staquorsin as an Infection Combating Agent

For the *in vivo* testing, the murine skin abscess model was adopted and a treatment regimen similar to the one described for savirin was followed (Sully et al., 2014). On the day prior to the experiment, the backs of female 6- to 8-week-old BALB/c mice were shaved. On day 0, the mice were weighed and distributed into two groups (five mice each). Bacterial cells were prepared as a suspension in 0.5% hydroxypropyl methylcellulose (HPMC). Fifty μl of the bacterial cell suspension (∼1 × 10^8^ CFU) were injected subcutaneously concurrently with 40 nmol of staquorsin, in 50 μl of 0.5% HPMC. For delayed delivery, 80 nmol of staquorsin in 100 μl of 0.5% HPMC were administered 24 and 48 h post-infection. The mice were kept in their cages, where they were given food and water *ad libitum*, and their weights were monitored daily. On day seven, the mice were euthanized using an overdose of 2,2,2-tribromoethanol in pyrogen-free saline. The skin lesions were aseptically removed. Bacterial burdens in the excised samples were assessed by homogenizing the tissue in 1 ml of normal saline, followed by serial dilution, plating on mannitol salt agar (MSA), and incubating at 37°C for 24 h for colony forming unit (CFU) counts.

### Statistical Analyses

GraphPad Prism v9 was used for statistical analyses. When appropriate, one-way ANOVA or two-way ANOVA were applied followed by Tukey’s multiple comparisons test. Otherwise, the performed test is described in the corresponding figure legend. The *p*-values ≤ 0.05 were considered significant.

## Results

### Spectroscopic Analyses Confirm the Staquorsin Structure

The staquorsin IR spectrum showed NH and SO_2_ bands at 3425, 3200, 1342, and 1157, respectively (Figure 1C). Its ^1^H NMR showed a singlet at 3.85 ppm corresponding to the OCH_3_ group along with two exchangeable signals at 8.50 and 10.67 ppm attributed to the 2 NH moieties. Additionally, the presence of two doublets confirmed the *para* substituted phenyl ring system (Figure 1D). These data confirmed the obtained product chemical structure.

### Staquorsin Inhibits the Agr System Both Phenotypically and Molecularly

**FIGURE 2:**
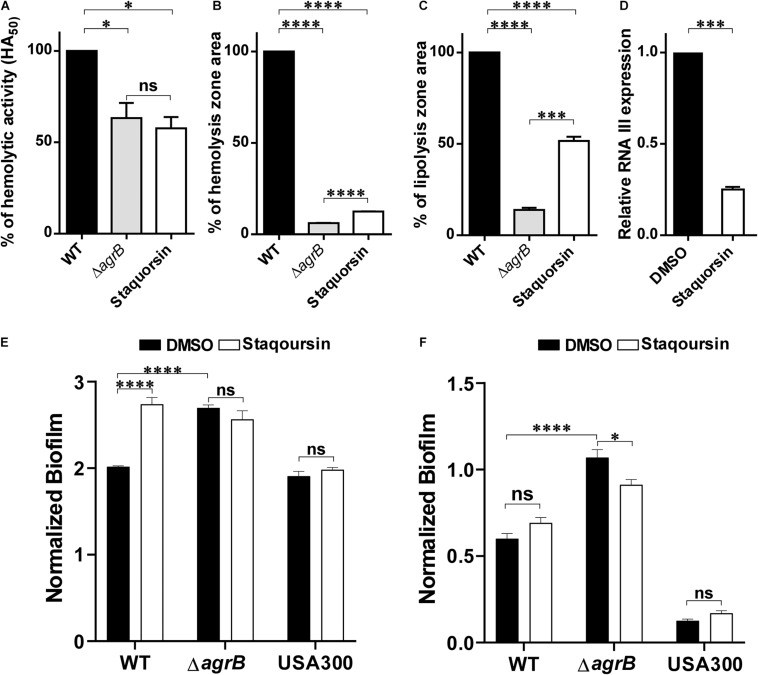
Staquorsin antagonizes the Agr system phenotypically and on the molecular level. The effect of staquorsin on some phenotypic Agr-related virulence activities **(A)** The alpha-hemolysin activity on rabbit RBCs as measured by the HA_50_ values. The HA_50_ of culture supernatants of cells grown in the presence of 40 μM staquorsin is presented as percentage of that of the WT grown in the absence of staquorsin. **(B)** The delta-hemolysin activity on sheep RBCs as measured by the area of the hemolysis zone. The area of the hemolysis zone produced by culture supernatants of cells grown in the presence of 200 μM staquorsin is presented as percentage relative to that of the WT grown in the absence of staquorsin. **(C)** The lipolytic activity as measured by the area of the zone of lipolysis on tributyrin agarose. The area of the lipolysis zone produced by culture supernatants of cells grown in the presence of 200 μM staquorsin is presented as percentage relative to that of the WT grown in the absence of staquorsin. In all of the above three charts, data of the Δ*agrB* strain was used as a negative control. Statistical analyses were performed by applying ordinary one-way ANOVA followed by Tukey’s multiple comparisons test. **(D)** Effect of staquorsin on the transcription of RNA III. The level of the RNA III in cells grown in the presence of 40 μM staquorsin was assessed and presented relative to the level in cells grown in its absence. Statistical analysis was performed by applying the unpaired *t*-test. **(E)** Effect of staquorsin on the biofilm formation. Strains were grown in the presence of either DMSO (black bars) or 40 μM staquorsin (white bars) and the formed biofilms were assessed by the crystal violet assay. The crystal violet reading at 595 nm was normalized by dividing it by the optical density of the planktonic culture at 600 nm. **(F)** Effect of staquorsin on the biofilm detachment. Biofilms was formed, then fresh TSB with either DMSO (black bars) or 40 μM staquorsin (white bars) was added for additional 24 h, then the biofilms were stained was crystal violet and processed as in **(E)**. Two-way ANOVA was applied followed by Tukey’s multiple comparisons test. In all the above charts, the data presented is the average of three independent experiments, the error bars represent the standard deviation, “ns” indicates non-significant statistical difference (*p-*value > 0.05), “^∗^” *p-*value ≤ 0.05, “^∗∗∗^” *p-*value ≤ 0.001, and “^*⁣*⁣**^” *p-*value ≤ 0.0001.

Since the Agr is known to control several virulence related phenotypes, staquorsin was tested for its ability to inhibit some of these phenotypes. First, staquorsin was successful in reducing the alpha-hemolytic activity of *S. aureus*. Staquorsin significantly (*p* < 0.05) lowered the HA_50_ of wild-type *S. aureus* almost to the same level of the Δ*agrB* mutant (Figure 2A). Assaying for the effect on the delta hemolytic activity, staquorsin significantly (*p* < 0.0001) reduced the area of the hemolysis zone in sheep blood agar by ∼88% compared to that of the wild-type strain (Figure 2B). This level of reduction was slightly lower than that caused by the Δ*agrB* mutant. Upon testing the effect on the lipolytic activity, staquorsin reduced the area of the lipolysis by more than 50% (*p* < 0.0001; Figure 2C). To confirm that the observed phenotypes are a reflection of a reduction in the RNA III transcript, which is the effector molecule of the Agr system, transcriptional analysis indicated that the growth in the presence of staquorsin reduced the level of transcription of the RNA III by 75% (*p* < 0.001) relative to its level in the presence of the vehicle (DMSO) (Figure 2D). The role of the Agr system in biofilms is well-established, as the repression of the *agr* enhances biofilm formation, while activation of the *agr* system is essential for the detachment of biofilm (Tan et al., 2018). As expected, the Δ*agrB* mutant formed more significant biofilm than its WT counterpart (Figure 2E). Growing the WT in the presence of 40 μM staquorsin, it formed a much better biofilm than that formed in the presence of equivalent amount of DMSO and very comparable that formed by the Δ*agrB* mutant (Figure 2E). Checking the effect of staquorsin on the biofilm formation in the USA300 strain, it only caused a slight, but non-significant increase (Figure 2E). Investigating the dispersal of formed biofilms, as expected the Δ*agrB* mutant showed less biofilm detachment than that seen in the WT (Figure 2F). Staquorsin managed to limit the dispersal of the WT biofilm better than just the DMSO, however this difference was not statistically significant (Figure 2F). Similar effect was also seen in strain USA300 (Figure 2F).

### Staquorsin Is Predicted to Bind to AgrA in a Way That Is Very Comparable to the Previously Described AgrA Inhibitor Savirin

**FIGURE 3:**
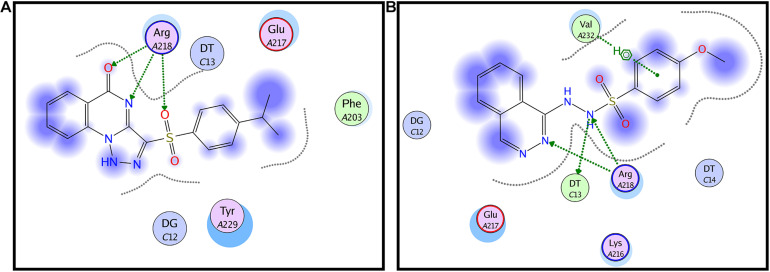
*In silico* docking of both savirin and staquorsin to the *S. aureus* AgrA. Two-dimensional diagrams of savirin **(A)** and staquorsin **(B)** docking pose interactions with the key amino acids in the AgrA binding site. The blue regions denote an area that can form hydrophobic interactions. Dotted arrows denote the formation of H bonds from the donor to the acceptor atoms. The diagrams were generated using the Molecular Operating Environment (MOE, 10.2008) software.

*In silico* docking studies indicated that staquorsin binds to the active site of AgrA in a very close manner to that of savirin. First, “savirin” was docked in the vicinity of the active site of the AgrA protein with energy score (S) = −7.12 kcal/mol with the ability to reproduce all the reported interactions with the key amino acids in the binding site; hydrogen bonding with Arg218 and π–π stacking with the Tyr229 (Sully et al., 2014) (Figures 3A,B). The validated setup was then used in predicting the ligands receptor interactions at the active site for staquorsin. Upon calculating the absolute binding energy score of these bindings, staquorsin showed a marginally lower score than that of savirin with −6.67 kcal/mol. A summary of the binding energy score, interacting amino acids and binding interactions of both compounds is presented in Table 1.

**TABLE 1:** Binding energy score, interacting amino acids, and binding interactions of savirin and staquorsin.

**Compound**	**Docking score (kcal/mol)**	**Involved amino acids**	**Binding interactions**
Savirin	−7.12	Arg218	Hydrogen bonding
		Tyr229	π–π stacking
Staquorsin	−6.67	Arg218	Hydrogen bonding
		Val232	Hydrophobic interactions

### Staquorsin Is Predicted to Have Better ADMET Properties Than Savirin

The *in silico* comparison between both savirin and staquorsin regarding the ADMET properties revealed that staquorsin has better water solubility and less lipophilicity than savarin and consequently, it has predicted higher GIT absorption. Furthermore, both compounds cannot pass blood brain barrier, therefore, it could be assumed that they will have no CNS side effects. Also, both do not bind to plasma glycoprotein making them bioavailable to exert their action. Finally, staquorsin is expected to have lower drug-drug interactions than savarin based on their deduced liver enzymes cytochrome P450 (CYP) inhibition data as savirin can inhibit two types of cytochrome P450 while staquorsin inhibit only one type. A summary of this comparison is presented in Table 2.

**TABLE 2:** Predicted lipophilicity, solubility, and pharmacokinetics of savirin and staquorsin.

**The property**	**Savirin**	**Staquorsin**
Lipophilicity (consensus log P)	3.27	2.00
Water solubility	Poorly soluble	Moderately soluble
GIT absorption	Low	High
BBB permeant	No	No
P-glycoprotein substrate	No	No
Cytochrome P450-1A2 inhibitor	No	Yes
Cytochrome P450-2C19 inhibitor	Yes	No
Cytochrome P450-2C9 inhibitor	Yes	No
Cytochrome P450-2D6 inhibitor	No	No
Cytochrome P450-3A4 inhibitor	No	No

### Staquorsin, Has Very Limited Effect on *S. aureus* Growth at Very High Concentrations as Opposed to Savirin

**FIGURE 4:**
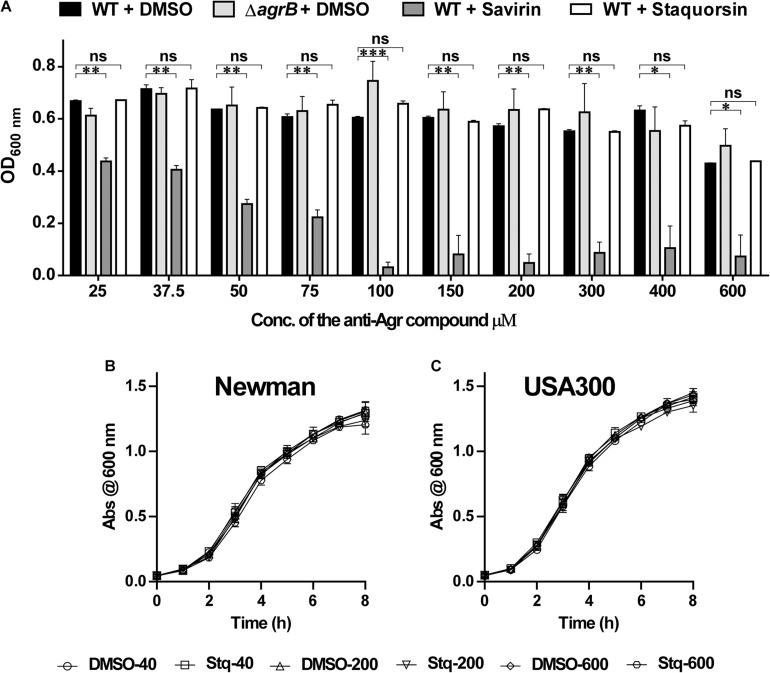
Staquorsin does not affect cell viability in very high concentrations as opposed to savirin. **(A)** Optical density of the bacterial culture after incubation for 24 h, in the presence of staquorsin (white bars), compared to the previously established quorum sensing inhibitor savarin (dark gray bars), the WT (black bars) and Δ*agrB* (light gray bars) grown in the presence of equivalent volumes of the vehicle (DMSO) used for dissolving the anti-Agr compounds. Effect of increasing concentrations of staquorsin on the growth kinetics of the MSSA strain Newman **(B)** and the MRSA strain USA300 **(C)**. Cell were grown in the indicated concentration of either staquorsin (Stq) or DMSO and the optical density at 600 nm was recorded at the indicated time points. The data presented is the average of three independent experiments and the error bars are the standard deviation. Statistical analyses were performed by applying ordinary one-way ANOVA followed by Tukey’s multiple comparisons test on the readings corresponding to each concentration. The “ns” indicates non-significant statistical difference (*p-*value > 0.05), “^∗^” *p-*value ≤ 0.05, “^∗∗^” *p-*value ≤ 0.01, and “^∗∗∗^” *p-*value ≤ 0.001.

To test the effect of the staquorsin on the growth of *S. aureus*, cells were grown in the presence of increasing concentrations of it. At the same time, the experiment was repeated using the savirin. As controls, both the wild-type and the *agr*-mutant were grown in the presence of equivalent amounts of the vehicle (DMSO). The OD at 600 nm after 24 h were recorded and plotted (Figure 4A). At concentrations as high as 600 μM, there was no significant difference between the extent of growth of *S. aureus* in the presence of either DMSO or staquorsin. On the other hand, with as low as 25 μM of savirin, there was a significant reduction in the growth extent of *S. aureus* (Figure 4A). Upon determination of the MIC for both compounds, savirin had an MIC of 100 μM while staquorsin MIC was >1600 μM. Therefore, staquorsin is a good inhibitor of the Agr system with minimal impact on the growth of *S. aureus* as opposed to savirin. In order to test whether staquorsin would affect the growth kinetics of *S. aureus*, two strains, Newman and USA300, were grown in different staquorsin concentrations and the growth was monitored over time. Upon testing strain Newman, staquorsin has no negative impact on either the growth rate or growth extent up to 600 μM (Figure 4B). Very similar effect was observed with USA300, where no differences were observed between the growth pattern in the presence of either staquorsin or the equivalent amounts of DMSO (Figure 4C). The lack of negative impacts was seen in the first 8 h of growth (Figures 4B,C) and even after 24 h (data not shown).

### *Staphylococcus aureus* Does Not Develop Resistance to Staquorsin Activity Despite Repeated Sequential Passage in Its Presence

**FIGURE 5:**
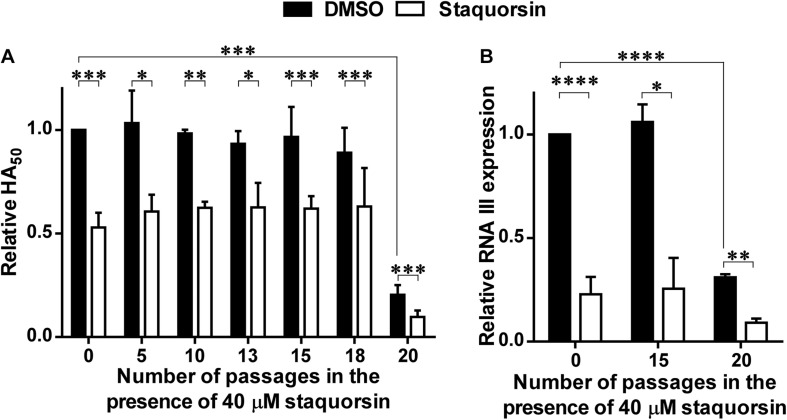
Staquorsin does not lose activity against *S. aureus* following repeated exposure. *S. aureus* cells were passed several times in the presence of 40 μM staquorsin then its effect on the HA_50_ of the culture supernatants **(A)** and the level of the RNA III **(B)** were assayed. In both charts, the data is presented relative to the results of the *S. aureus* cells which were not exposed to staquorsin (the first black column). The data presented is the average of three independent experiments and the error bars are the standard deviation. Statistical analyses were performed by applying an unpaired *t*-test for each pair representing the readings after the indicated number of passages that the cells were exposed to. The “^∗^” indicates a *p-*value ≤ 0.05, “^∗∗^” *p-*value ≤ 0.01, “^∗∗∗^” *p-*value ≤ 0.001, and “^****^” *p-*value ≤ 0.0001.

To address the possibility of resistance development to staquorsin, the wild-type *S. aureus* was passed sequentially 20 times in the presence of 40 μM staquorsin. Initially, the HA_50_ was reduced by staquorsin by ∼50% as earlier seen in Figure 2A, a comparable level of reduction was seen after 5, 10, 13, 15, 18, and 20 passages (Figure 5A). Interestingly, the wild-type strain lost part of its hemolytic activity after the 20th passage. This could be due a decrease in the activity of the Agr system after these multiple passages. However, staquorsin managed to reduce this activity by the same extent (∼50%) as observed in the previous passages (Figure 5A). On the molecular level, the effect of staquorsin on *S. aureus* RNA III level was also maintained even after repeated passages; whereas after 15 passages, staquorsin had similar activity. However, after 20 passages, although the level of RNA III transcription in the positive control was lowered, staquorsin has continued to reduce the RNA III transcription level, where RNA III concentration fell to less than one third that of the positive control (*p* < 0.001; Figure 5B). This confirms that the observed reduction in the hemolytic activity seen in the strain obtained after the 20th passage is due to decrease in the Agr activity. The wild-type, passed in subinhibitory concentration of erythromycin (0.8 μg/ml), required only 8 passages for the development of resistance. The MIC had risen from 1 to 4 μg/ml by the 8th passage and to 16 μg/ml by the 16th passage.

### Staquorsin Inhibition of the Agr-System Promotes Skin Infection Management

**FIGURE 6:**
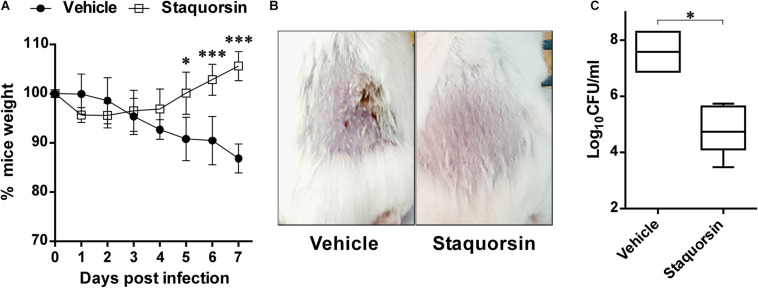
Staquorsin is effective in controlling *S. aureus* skin infection. **(A)** Monitoring the weights of the mice throughout the course of the infection. The weights of mice were determined every 24 h and they are presented as percentages of the weights at day 0. The data presented is the average of the readings of the mice in each group; treated with the vehicle (closed circles) and staquorsin (open squares), and the error bars represent the standard deviation. Statistical analyses were performed by applying an unpaired t test for each pair representing the readings at the indicated time point. **(B)** Photographs of a representative mouse of each group (treated with vehicle vs staquorsin). The images were taken on day seven post-infection. **(C)** Box plots of the bacterial loads in the skin lesions of mice infected with *S. aureus* and treated with either the vehicle or staquorsin. The data is presented as Log_10_CFU/ml obtained following plating the homogenates of the skin lesions excised from the infected mice. The whiskers span the difference between the minimum and maximum readings; the horizontal bar represents the mean. The “^∗^” indicates a *p-*value ≤ 0.05, and “^∗∗∗^” *p-*value ≤ 0.001.

The potential ability of staquorsin, to modulate *S. aureus* infection, was assessed *in vivo* by adopting the murine skin infection model. Prior to conducting the animal experiment, cytotoxicity assays were performed to ensure the safety of this compound. The HSF cells showed viability up to 95.06% ± 0.26 following incubation with 100 μM of staquorsin for 24 h (Table 3). Keeping in mind that most of the observed *in vitro* phenotypes in the current study was detected with just 40 μM, this would argue that staquorsin is predicted to be very safe upon contact with mammalian cells. All mice infected with *S. aureus* started to lose weight. Those treated with just the vehicle continued to lose weight throughout the whole course of the infection (Figure 6A). On the other hand, mice given staquorsin started to attain significant weight gain starting from day 5 post-infection (Figure 6A). Mice given the vehicle only, showed inflamed skin with abscess formation and ulceration. Whereas, mice treated with staquorsin showed signs of healing, evident by less inflammation and the lack of ulceration and abscess formation by day seven (Figure 6B). Upon evaluating the bacterial loads in the skin lesions, the mice treated with staquorsin showed a significant reduction of almost three log cycles when compared to those in the mice treated with the vehicle only (Figure 6C).

**TABLE 3:** Results of the cytotoxicity assay on human skin fibroblasts.

**Staquorsin concentration (μM)**	**% Viability (mean ± SD)**
0	100
0.01	99.37 ± 0.26
0.1	97.21 ± 0.17
1	97.14 ± 0.26
10	95.68 ± 0.94
100	95.06 ± 0.26

## Discussion

In 2014, the WHO announced antimicrobial resistance as a public health threat that requires global action. Searching for novel antimicrobials to overcome these resistant pathogens is not an ideal solution. As the development of resistance to new agents typically occurs at much faster rates than expected, in addition to the scarcity of the new compounds developed. Finding new strategies to tackle resistant organisms is of great concern.

Anti-virulence agents offer a promising alternative that can help in combating resistant bacteria (Totsika, 2016). Targeting *S. aureus* Agr-system mediated QS has proven efficient in reducing its virulence allowing the host defense to eradicate different *S. aureus* infections (Sully et al., 2014).

In this work, a newly synthesized compound was tested for its *S. aureus* anti-virulence capabilities especially those related to the Agr system. The developed compound was compared to the previously described *S. aureus* virulence-inhibitor savirin (Sully et al., 2014). The Agr system is a master regulator of QS which in turn controls diverse virulence factors of *S. aureus* (Cheung et al., 2004; Queck et al., 2008). One component of the Agr system is AgrA, which is a response regulator that upon activation binds to either the P2 or the P3 promotors. Binding to the former, activates a positive feedback loop to amplify the production of the components of the system, while binding to the latter induces the production of the RNA III (Koenig et al., 2004). RNAIII is a small regulatory RNA molecule that blocks the translation of the toxins repressor (Rot), leading to upregulation of multiple virulence factors (Boisset et al., 2007; Le and Otto, 2015).

Staquorsin demonstrated several phenotypes that linked it to having an anti-Agr activity such as down-regulation of the alpha and delta-hemolytic activities, lipolytic activity, the down-regulation of the RNA III transcript, enhancing biofilm formation, and blocking the biofilm dispersion (Figure 2). In addition, the *in silico* analysis indicated that staquorsin docks in the AgrA C-terminal at a critical position for proper folding and DNA binding (Sun et al., 2012), with a slightly lower absolute binding score than savirin. Inhibition of the *agr* operon through AgrA is highly advantageous, as AgrA sequence is conserved among the four types of the Agr system (Tan et al., 2018).

These results provoked the question, what advantage would staquorsin have over the already described QS inhibitor, savirin. A key feature sought in anti-virulence therapies is their lack of negative impact on the viability of the pathogen, leading to a decrease in the frequency of resistance development (Rasko and Sperandio, 2010; Heras et al., 2015; Rampioni et al., 2017; Totsika, 2017; Fleitas Martinez et al., 2019; Amer et al., 2021). In the work described by Sully and co-workers, they showed that savirin has no effect on the viability of *S. aureus* in a concentration up to 6.3 μM, however, upon testing the anti-Agr activity of this compound they routinely used a concentration of 5 μg/ml (13.6 μM) which is more than double the concentration indicated to have no effect on the viability (Sully et al., 2014). Accordingly, we wanted to test whether higher concentrations of savirin would have any impact on the viability of *S. aureus*. Upon testing less than double the concentration used by Sully and co-workers (25 μM) there was a significant reduction (∼35%) in the extent of growth (Figure 4A). Higher concentrations of savirin led to more reduction in the extent of the growth of *S. aureus*. On the contrary, staquorsin did not demonstrate significant impact on the growth of *S. aureus* at concentrations up to 600 μM. Accordingly, staquorsin comes with a great advantage over savirin with its ability to exert much lesser pressure on the microbial cell by exerting minimal effect on its viability. Moreover, no negative impact was seen on the growth kinetics in a concentration up to 600 μM.

The lack of selective pressure of staquorsin was confirmed by the sequential passage of *S. aureus* 20 times at a relatively high concentration (40 μM) and yet there was no development of resistance. This lack of resistance development was confirmed both phenotypically and molecularly. It is worth mentioning that the serial passage of *S. aureus* in the presence of staquorsin resulted in a strain after the 20th passage that has lower basal Agr activity as demonstrated by lower RNA III transcript and lower HA50. This phenomenon has been seen before by Somerville and co-workers, where the *in vitro* serial passage of *S. aureus* led to changes in physiology, virulence factors production, and *agr* nucleotide sequence (Somerville et al., 2002). Yet despite of this observed decrease, growth of this strain in the presence of staquorsin demonstrated a further decrease in the Agr activity indicating the staquorsin is still active on its target despite its lowered basal activity. On the other hand, resistance developed rapidly for erythromycin as the MIC quadrupled after the 8th passage.

Resistance to QS inhibitors may develop *in vivo*. In the case the *agr* system, mutations causing inhibitor resistance can result from *agr* operon dysfunctional mutants, which are less able to colonize and cause infection to the host. They are also incapable of spreading efficiently between patients (Shopsin et al., 2010). However, a recent review of literature and meta-analysis carried out by Lee and co-workers concluded that dysfunctional *agr* could influence the outcome of invasive *S. aureus* infections depending on conditions like the oxacillin susceptibility and the site of infection (Lee et al., 2020). This was observed prominent in MRSA and pneumonia where the dysfunctional *agr* was generally associated with unfavorable clinical outcomes.

To assess the efficacy of staquorsin *in vivo*, it was tested in the skin infection model as most MRSA infections are of this type and the role of the Agr system in establishing infection is confirmed (Salam and Quave, 2018). Staquorsin was able to reduce the signs of infections like weight-loss and the extent of inflammation in the tissues. In addition, bacterial loads in the skin lesions were significantly lowered.

In conclusion, the current study models staquorsin as a promising *S. aureus* QS inhibitor. It demonstrated extraordinary efficiency to strip down *S. aureus* of a substantial part of its weaponry. Further study is nevertheless required to confirm its possible action on different *agr* types in pathogenic *S. aureus* strains. Also, for its ability to clear different infection types, other than the skin infection.

## Data Availability Statement

The original contributions presented in the study are included in the article/supplementary material, further inquiries can be directed to the corresponding author/s.

## Ethics Statement

The animal study was reviewed and approved by Research Ethics Committee of the Faculty of Pharmacy, Cairo University with number MI(1682).

## Author Contributions

All authors have contributed to the design of the experiments, collecting the data, analyzing the results, drafting the manuscript, and reviewing the manuscript prior to submission.

## Conflict of Interest

The authors declare that the research was conducted in the absence of any commercial or financial relationships that could be construed as a potential conflict of interest.
